# A Case of Tension Pneumothorax After Diverticular Rupture During Diagnostic Colonoscopy

**DOI:** 10.7759/cureus.13003

**Published:** 2021-01-30

**Authors:** Badri Kobalava, Dima Chachkhiani, Nana Turava, Giorgi Giorgobiani

**Affiliations:** 1 Surgery Department #3, Faculty of Medicine, Tbilisi State Medical University, Tbilisi, GEO; 2 Surgery Division, Aversi Clinic, Tbilisi, GEO; 3 School of Medicine, New Vision University, Tbilisi, GEO; 4 Radiology Division, Aversi Clinic, Tbilisi, GEO

**Keywords:** intestinal perforation, diverticular perforation, colonoscopy complications, secondary pneumothorax

## Abstract

Colonoscopy is routinely used for the diagnosis and treatment of colorectal diseases. Bowel perforation is a rare but severe complication that significantly increases the morbidity and mortality. Tension pneumothorax is an uncommon complication of colonic perforation. We present a case of the successful treatment of a patient with tension pneumothorax, following colonoscopy, by using tube thoracostomy and Hartman-type resection of the rectosigmoid junction and proximal sigmoid. Surgeons, anesthesiologists, and endoscopists should consider the possibility of pneumothorax as a rare complication of colonoscopy. Early detection and urgent treatment is the key to successful management.

## Introduction

Colonoscopy is the gold standard for diagnosing colorectal diseases. It is a safe procedure associated with low mortality (0.0029%) [1], and the most common major complication is bleeding (0.164%) [2]. Perforation is a rare but life-threatening complication that significantly increases morbidity (36%) and mortality (7%) [3].

## Case presentation

A 73-year-old Caucasian woman with a two-week history of lower abdominal pain was sent to gastroscopy and colonoscopy by a gastroenterologist. The patient had no symptoms of acute abdomen. Following colon preparation using sodium dihydrogen phosphate, endoscopy was started under intravenous anesthesia with spontaneous ventilation.

Esophagogastroduodenoscopy lasted 3.5 minutes and was completed uneventfully. The colonoscope was then advanced to the proximal sigmoid colon and several diverticulae were found in this area. Because of the colonic tortuosity it was difficult to advance the scope further. More insufflation was employed to straighten the kinking without usage of the high-pressure insufflation or compressed air, but after about 15 minutes of unsuccessful trial, the procedure was abandoned.

During recovery, the anesthesiologist detected subcutaneous emphysema at the cervical and upper thoracic area and increasing respiratory distress. Lung sounds were muffled bilaterally, mostly on the right. Tachycardia (125 beats per minute) and hypotension developed, and vasopressor support was started. Despite 100% oxygen administration with a mask, saturation decreased gradually. The trachea was intubated by quick protocol, and the patient was transferred to the intensive care unit.

**Figure 1:**
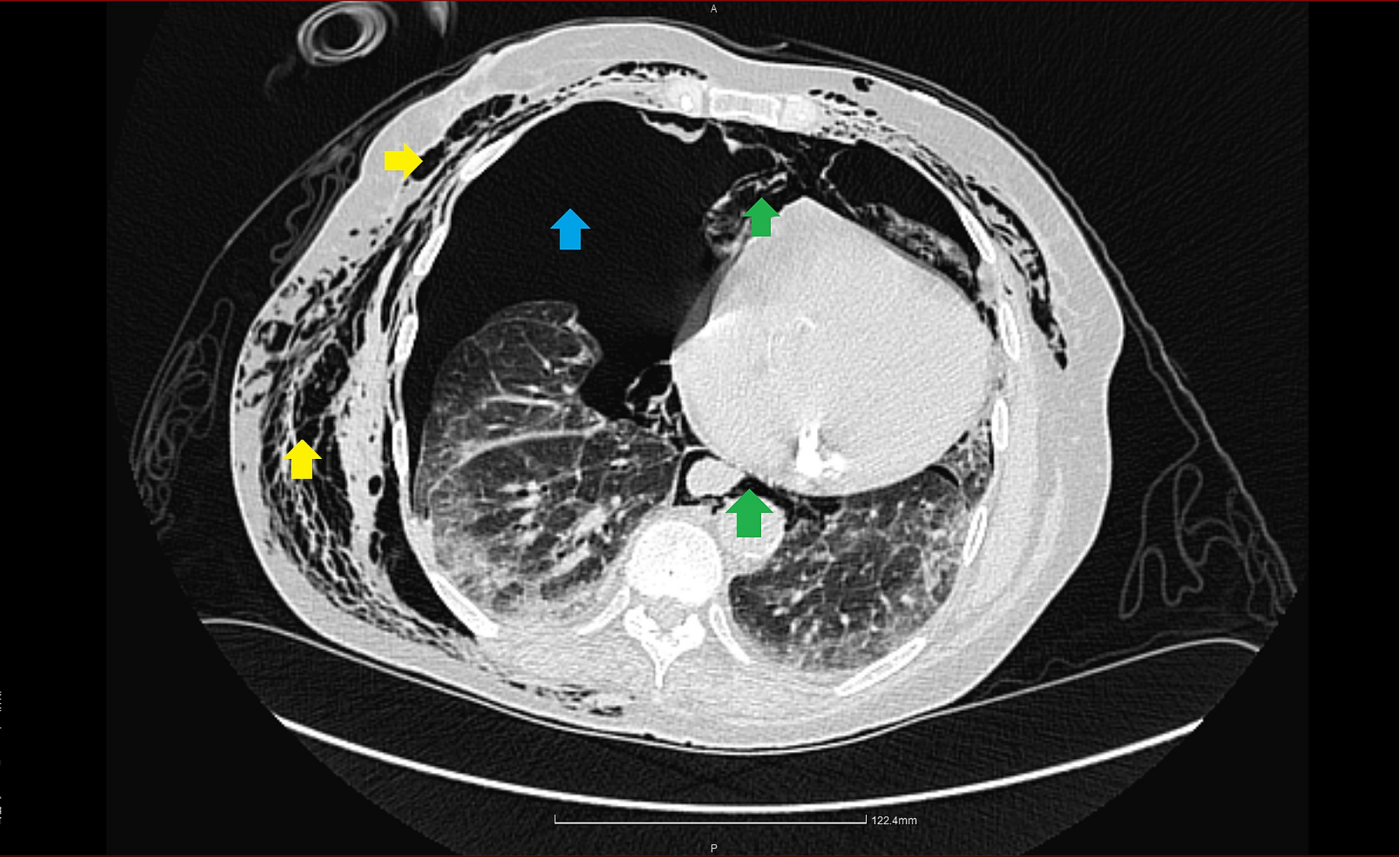
Right-sided tension pneumothorax. Blue arrow: pneumothorax; green arrows: pneumomediastinum; yellow arrows: subcutaneous emphysema.

**Figure 2:**
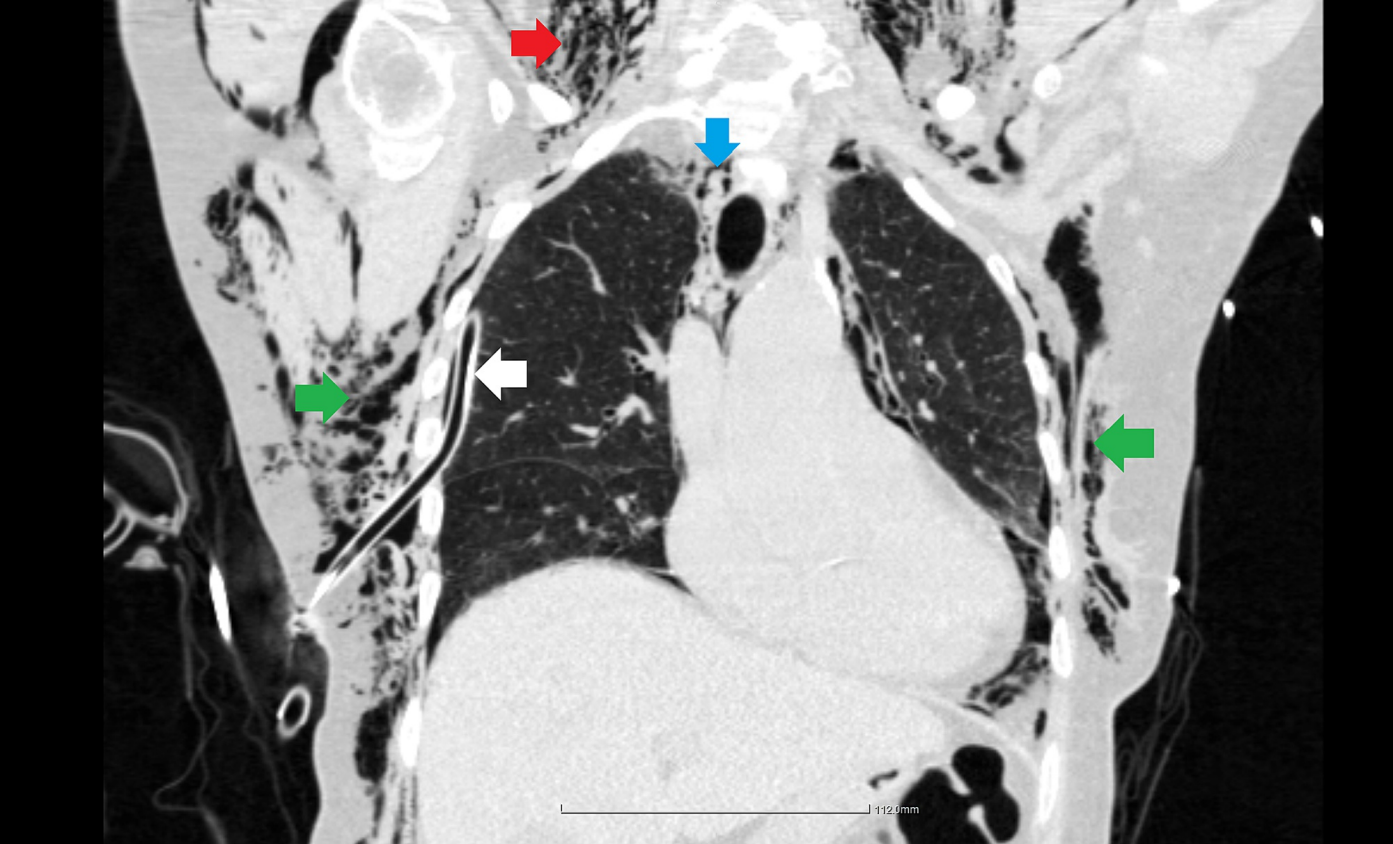
Right-sided tube thoracostomy. White arrow: thoracic drain; blue arrow: pneumomediastinum; green arrows: subcutaneous emphysema; red arrow: cervical emphysema

**Figure 3:**
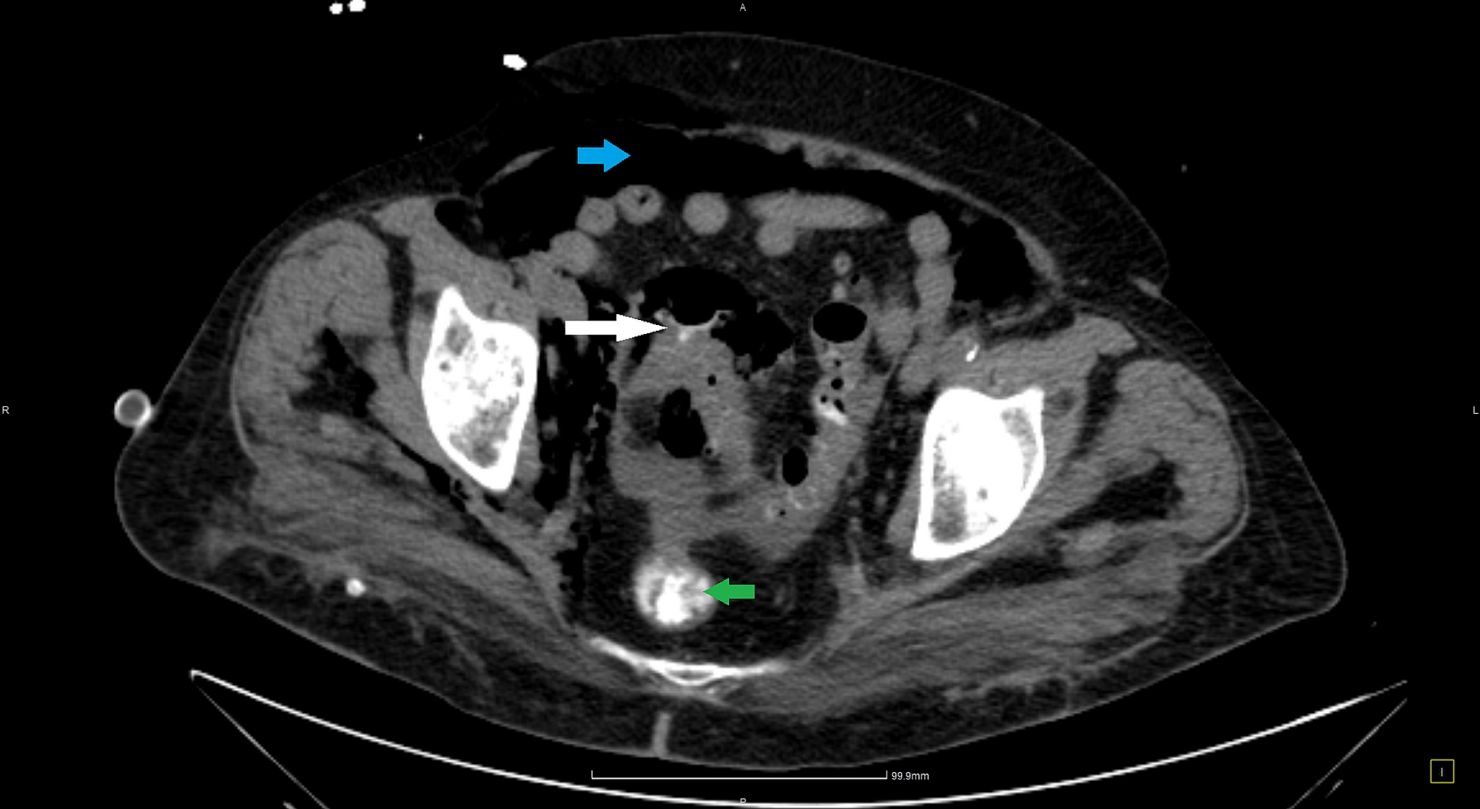
CT with rectal contrast. Green arrow: contrast in rectum; white arrow: extraluminal contrast; blue arrow: pneumoperitoneum. CT, computed tomography

No respiratory sounds were detected on the right side. After stabilization of the hemodynamic parameters, chest and abdominal computed tomography (CT) was done, which confirmed subcutaneous emphysema, bilateral pneumothorax, right-sided tension pneumothorax, pneumomediastinum, and pneumoperitoneum (Figure 1). A tube thoracostomy was performed. After the re-expansion of the lung, no continuous air leakage was detected from the thoracic tube (Figure 2). Hemodynamic and respiratory parameters improved quickly. However, significant abdominal distension, rigidity, and tenderness were found. CT with rectal contrast revealed colonic diverticular disease and extraluminal contrast adjacent to the distal sigmoid colon (Figure 3). At laparotomy, the mesentery of the distal sigmoid colon was perforated adjacent to the tenia mesenterica. A hole in the sigmoid mesentery, contaminated with feces, was observed. Hartman-type resection of the rectosigmoid junction and distal sigmoid colon was performed, and a colostomy was created at the left flank.

On postoperative day (POD) 1, the trachea was extubated. Oral liquids were given on POD2. On POD3, the patient was transferred to wards and ambulation was started. Soft food was initiated the next day. Finally, the patient was discharged on POD6. The pathology confirmed the rupture of the diverticulum at the mesenteric side of the sigmoid colon.

Two months after the surgery, the patient was hospitalized for acute heart failure. She complained of fatigue, dyspnea, and abdominal discomfort. No signs of infection were found. The colostomy functioned well. The pericardial and small bilateral pleural effusions were treated without intervention. Twelve weeks after the surgery, the patient had no complaints. No significant abnormality was revealed on complete blood count, abdominal ultrasound, or chest X-ray.

## Discussion

The incidence of the perforation is <1:500 for all colonoscopies and <1:1,000 for screening procedures [4]. Perforations commonly occur after endoscopic minimally invasive interventions [5] rather than during diagnostic/screening colonoscopy when it is caused by mechanical injury or barotrauma as a result of high-pressure insufflation (>80 mmHg) [6]. The risk of injury increases in case of an underlying colonic lesion such as tumor, diverticulum [7], or inflammatory bowel diseases [8]. The most common site of colonic perforation is the proximal sigmoid colon [9]. Advanced age (>65 years) and female gender are the leading risk factors for iatrogenic colonoscopy perforations [10]. Females have inherently longer colons and deep pelvises, which makes colonoscopy more difficult. A wide and rounded pelvis predisposes the patient to the redundant sigmoid colon and the need for higher pressure insufflation [11].

Tension pneumothorax is an uncommon complication of colonic perforation. Publications dedicated to this problem are rare and only 34 cases have been reported in PubMed. Spontaneous pneumothorax is the accumulation of the air in the pleural cavity without trauma or medical intervention on the thoracic wall or intrathoracic organs. It can be primary that develops without any clinically apparent preexisting lung illness or secondary that complicates underlying lung disease. Our patient had no history of the lung condition and obviously no thoracic trauma. Therefore, we suggested the diagnosis of primary spontaneous pneumothorax. The reason for this condition is the rupture of the subpleural bleb or emphysematous bulla in 80-100% of cases and the best method to diagnose this condition is thoracic CT [12]. In the presented patient, the chest CT did not reveal any pulmonary reason for the pneumothorax neither before nor after thoracostomy. Besides, the majority of the patients with primary spontaneous pneumothorax are boys or young men [13]. Because of the history of recent colonoscopy, presence of the pneumoperitoneum and pneumomediastinum, and developing signs of the peritonitis, we suggested colonic damage associated with endoscopy. Due to the curiosity of the complication, we performed CT with rectal contrast to evidence the perforation by detection of the extraluminal contrast.

The perforation of the sigmoid colon can occur inside the mesentery or into the peritoneal cavity. Therefore, two routes of gas transfer are possible from the colonic lumen to the pleural space: one along the loose connective tissue connections and the second across the diaphragm after the creation of the pneumoperitoneum.

First, perforation into the mesosigmoid can result in the spread of the gas into the retroperitoneal space. Intramesenteric connective tissue is a continuation of the retroperitoneum. The anatomic connection between the retroperitoneal space, mediastinum, and cervix is well known [14]. During embryonic development, the coelomic cavity is divided into the thoracic and abdominal spaces after the formation of the diaphragm. Subperitoneal and subpleural spaces still communicate through hiatuses and gaps forming the cervicothoracic continuum [15]. In addition, the midline gap in the diaphragm, between its sternal parts and two paramedian sternocostal gaps of Morgagni, provides the attachment of the endothoracic and endoabdominal fasciae. In those areas, passage of the gas from retroperitoneal to mediastinal space is possible [14].

Second, the pressure gradient and small congenital gaps in the tendinous part of the diaphragm generate the transfer of air from the peritoneal cavity into the pleural space [16]. CT of our patient demonstrated only a small amount of air in the peritoneal cavity. Without the tension pneumoperitoneum, passage of the air across the diaphragm is less likely. In some publications on post-colonoscopic pneumothorax and pneumoperitoneum, the perforation was not found, but the patient could be successfully managed without surgery [17]. Such cases can be explained by transfer of the air from the retroperitoneum to the peritoneal cavity after intramesenteric perforation. In most reported cases, bowel perforation was obvious, and the treatment was primary closure or diversion.

We consider that, in our case, air insufflation during colonoscopy caused intramesenteric rupture of the sigmoid diverticulum. Air propagated along the mesentery first to the retroperitoneal space, followed by to the mediastinum and pleural space. Later, rupture of the mesenteric peritoneum caused intraabdominal spread of the air and fecal peritonitis. Such a scenario is well-known in the context of acute colonic diverticulitis.

The main treatment of colonic perforation is surgery, but in selected patients, endoscopic [18] or conservative therapy is feasible [19]. Surgical options include primary repair, wedge resection, and colonic resection with or without colostomy. According to the World Society of Emergency Surgery guidelines for the management of iatrogenic colonoscopy perforation [20] Hartmann-type resection was performed based on the following criteria: devitalized edges of the perforation (ruptured diverticulum), peritoneal contamination because of the imperfect bowel preparation, avulsion of the mesentery in the area of the damage, advanced age, and deteriorated general state of the patient caused by recent tension pneumothorax.

## Conclusions

Bowel perforation is a rare but severe complication of colonoscopy that significantly increases the morbidity and mortality rates of such diseases. The described case demonstrates an unusual consequence of colonic diverticular rupture during diagnostic endoscopy. Surgeons, anesthesiologists, and endoscopists must keep in mind the possibility of pneumothorax as a rare complication of colonoscopy, especially if the risk is elevated (female patient, advanced age, endoscopic intervention). Early detection and urgent treatment is the key to successful management.
